# Assessing spatio-temporal variability of risk surfaces using residential history data in a case control study of breast cancer

**DOI:** 10.1186/1476-072X-4-9

**Published:** 2005-04-12

**Authors:** Daikwon Han, Peter A Rogerson, Matthew R Bonner, Jing Nie, John E Vena, Paola Muti, Maurizio Trevisan, Jo L Freudenheim

**Affiliations:** 1 Department of Social and Preventive Medicine, University at Buffalo, Buffalo, NY 14214 USA; 2 Department of Geography and National Center for Geographic Information and Analysis, University at Buffalo, Buffalo, NY 14261 USA; 3 Department of Biostatistics, University at Buffalo, Buffalo, NY 14214 USA; 4 Department of Epidemiology and Biostatistics, University of South Carolina, Columbia, SC 29208 USA

## Abstract

**Background:**

Most analyses of spatial clustering of disease have been based on either residence at the time of diagnosis or current residence. An underlying assumption in these analyses is that residence can be used as a proxy for environmental exposure. However, exposures earlier in life and not just those in the most recent period may be of significance. In breast cancer, there is accumulating evidence that early life exposures may contribute to risk. We explored spatio-temporal patterns of risk surfaces using data on lifetime residential history in a case control study of breast cancer, and identified elevated areas of risk and areas potentially having more exposure opportunities, defined as risk surfaces in this study. This approach may be more relevant in understanding the environmental etiology of breast cancer, since lifetime cumulative exposures or exposures at critical times may be more strongly associated with risk for breast cancer than exposures from the recent period.

**Results:**

A GIS-based exploratory spatial analysis was applied, and spatio-temporal variability of those risk surfaces was evaluated using the standardized difference in density surfaces between cases and controls. The significance of the resulting risk surfaces was tested and reported as *p*-values. These surfaces were compared for premenopausal and postmenopausal women, and were obtained for each decade, from the 1940s to 1990s. We found strong evidence of clustering of lifetime residence for premenopausal women (for cases relative to controls), and a less strong suggestion of such clustering for postmenopausal women, and identified a substantial degree of temporal variability of the risk surfaces.

**Conclusion:**

We were able to pinpoint geographic areas with higher risk through exploratory spatial analyses, and to assess temporal variability of the risk surfaces, thus providing a working hypothesis on breast cancer and environmental exposures. Geographic areas with higher case densities need further epidemiologic investigation for potential relationships between lifetime environmental exposures and breast cancer risk. Examination of lifetime residential history provided additional information on geographic areas associated with higher risk; limiting exploration of chronic disease clustering to current residence may neglect important relationships between location and disease.

## Background

In a recent analysis of breast cancer by New York State's Department of Health, a breast cancer cluster in the Western New York area was identified [1]. One of the objectives of such disease mapping is to generate hypotheses by identifying spatial patterns so that causal processes may be evaluated further by more rigorous epidemiologic study. Spatial analyses have played a valuable role in explaining different health outcomes and in uncovering environmental causes of disease [2,3]. Residential locations at the time of diagnosis have generally been used in these exploratory spatial analyses [4,5]. Disease mapping has been increasingly used to identify spatial patterns with the aid of Geographic Information Systems (GIS) and exploratory spatial analysis tools, and has been a valuable tool for studies of geographic and environmental epidemiology, especially when the causes of disease and their determinant processes are unknown [6-8]. In particular, there has been recent interest in the use of kernel density estimation methods in epidemiologic studies. Density estimation methods have been used to smooth out noise based on functions of the data in surrounding areas and to overcome problems associated with traditional disease mapping [9,10].

Previous studies using exploratory spatial analyses, however, have been based on either residence at the time of diagnosis or current residence, and only a few recent studies have examined disease risk using information on lifetime residence [11,12]. For chronic disease, there is increasing evidence that lifetime exposures may be more relevant in understanding disease etiology. For breast cancer in particular, several of the well established risk factors (age at menarche, age at first birth) are from early life. There is now evidence that childhood and even *in utero *exposures may affect risk [13]. To examine disease clustering, lifetime cumulative exposures or exposures at critical times in a life course may be more strongly associated with risk for breast cancer than exposures from any one time period, especially the recent period.

In this study, we explored spatio-temporal patterns of risk surfaces using data on lifetime residential history in a case control study of breast cancer. We had previously identified geographic clustering of residence at critical points in early life in relation to breast cancer risk [14]. Here we focused on lifetime cumulative exposure in relation to the disease risk. Risk surfaces were created based on the relative densities of cases and controls – this indicated areas with higher case density as being areas with higher breast cancer risk, thus identifying areas potentially having more exposure opportunities. We used residence as a proxy for potential environmental exposures, conducted exploratory spatial analyses of breast cancer, and produced risk surface maps using information on lifetime residence to identify areas with high breast cancer incidence. In particular, we assessed spatio-temporal variability of risk surfaces using the standardized difference in case and control density, and evaluated the potential use of different kernel density estimation methods in applying them to epidemiologic data.

## Results

Descriptive characteristics of study participants by menopausal status are presented in Table 1, and characteristics of lifetime residential history for breast cancer cases and controls are summarized in Table 2. One-fourth of the study participants had at least one previous residence outside the study area, and these were excluded from the analysis. For those residences in the study area, we found that cases were somewhat more mobile, averaging 5.8 and 5.4 residences for pre- and postmenopausal participating cases, compared to 4.9 and 5 residences for pre- and postmenopausal participating controls, respectively.

**Table 1:** Descriptive characteristics of study participants (Mean ± Standard Deviation): WEB Study, 1996–2001.

	Premenopausal		Postmenopausal	
	Case (n = 325)	Control (n = 610)	Case (n = 841)	Control (n = 1495)

Age (years)	44.9 ± 4.6	44.1 ± 4.6	63.0 ± 8.5	63.4 ± 8.9
Education (years)	14.0 ± 2.3	14.2 ± 2.2	13.3 ± 2.6	13.0 ± 2.3
Parity	1.9 ± 1.3	2.0 ± 1.3	3.0 ± 1.9	2.5 ± 1.8
Age at menarche (years)	12.5 ± 1.6	12.6 ± 1.6	12.6 ± 1.6	12.8 ± 1.7
Age at first birth (years)	25.0 ± 5.1	25.8 ± 4.8	23.8 ± 4.7	23.5 ± 4.3
Recent BMI (kg/m^2^)	26.7 ± 6.6	27.2 ± 6.8	28.9 ± 6.1	28.4 ± 6.4
Benign breast disease (yes)	37%	21%	33%	22%
Relative with breast cancer (yes)	21%	10%	20%	14%

**Table 2:** Descriptive characteristics of lifetime residential history for breast cancer cases and controls: WEB Study, 1996–2001;

	Erie and Niagara (n = 15487)		Outside (n = 4752)		Total (n = 20240)	
	
	Case	Control	Case	Control	Case	Control
	Premenopausal					
Total numbers of residences in lifetime	1661	2767	432	948	2093	3715
Average numbers of residences per participant	5.8	4.9	3.2	3.4	5.0	4.4
Average years in each residence* (Mean ± SD)	5.6 ± 6.0	6.2 ± 6.6	4.3 ± 5.4	3.9 ± 4.9	5.3 ± 5.9	5.5 ± 6.3

	Postmenopausal					
Total numbers of residences in lifetime	4217	6842	1290	2082	5508	8924
Average numbers of residences per participants	5.4	5.0	3.5	3.2	4.8	4.4
Average years in each residence* (Mean ± SD)	6.9 ± 7.3	7.3 ± 7.9	4.8 ± 5.6	5.2 ± 6.3	6.3 ± 7.0	6.7 ± 7.6

* Excludes residences with missing data for length of residence.

**Figure 1:**
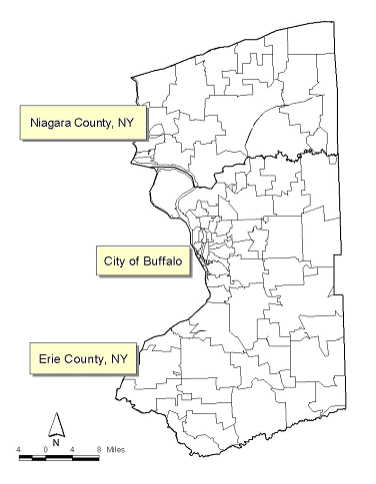
Map of study area: Erie and Niagara counties with zip-code boundaries.

**Figure 2:**
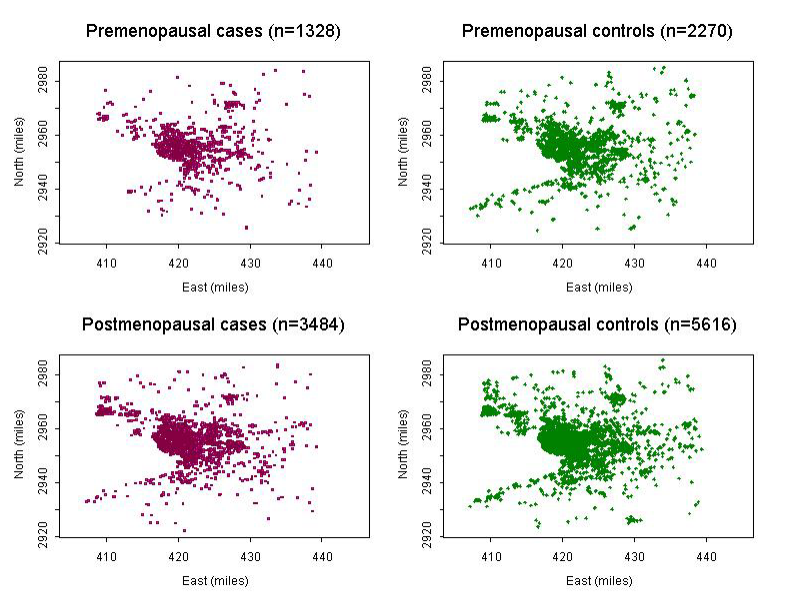
**Geographic distribution of breast cancer in Western New York; **Shown are all residential locations of breast cancer cases and controls by menopausal status included in the analysis. One dot indicates each residential location. The rectangular region was used as an approximate boundary of the study area instead of actual county boundary in Figure 1. East (x) and north (y) coordinates in projected Universal Traverse Mercator (UTM) miles.

We constructed lifetime cumulative risk surfaces to represent exposure opportunities in lifetime because cumulative exposures may be a more accurate indicator of potential environmental exposures related to breast cancer risk. Figure 1 shows a boundary map of Erie and Niagara counties, while Figure 2 depicts geographic patterns of lifetime residential locations for breast cancer cases and controls by menopausal status. We used the rectangular region as an approximate boundary of the study area to protect individuals' confidentiality. In the study area, there are 4,812 lifetime residential locations for cases (1,328 pre-menopausal and 3,484 post-menopausal residences), and there are 7,886 lifetime residential locations for controls (2,270 pre-menopausal and 5,616 post-menopausal residences).

**Figure 3:**
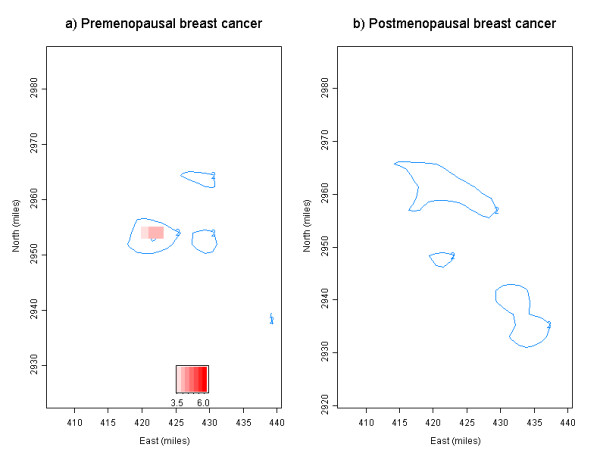
**Risk surfaces of pre- and postmenopausal breast cancer using standardized difference; **Areas with standardized difference greater than 2 SD are portrayed as contours of 2, and areas exceeding critical value of 3.56 as red images. The rectangular region was used as an approximate boundary of the study area instead of actual county boundary in Figure 1. East (x) and north (y) coordinates in projected UTM miles.

We evaluated spatial patterns of risk surfaces based on the geographic distribution of lifetime residences in Figure 2. Risk surfaces based on the standardized difference between case and control densities were obtained, and areas with relatively higher case density were identified by menopausal status in Figure 3. In the figure, areas with difference greater than 2 standard deviations (SD) were portrayed as contours and areas exceeding critical values were portrayed as red images. Testing for significance was performed and reported as *p*-values. Those areas with standardized difference greater than 2 SD were quite different between pre- and postmenopausal breast cancer, although ranges of standardized the difference were similar; -6.37 to 4.43 for pre-menopausal, and -6.57 to 3.39 for postmenopausal breast cancer, respectively. There were about 29 rectangular grids in those areas greater than 2 SD for premenopausal, while about 59 grids for postmenopausal breast cancer. Further, the statistical significance of areas must be assessed in light of the fact that multiple areas are tested; these are statistically significant if the difference in density exceeds the critical value of 3.56 at α = 0.05 (determined by simulation, where random labelling of cases and controls is carried out). There is one small geographic area of special interest for pre-menopausal residences in the central and upper region of the city. When these are compared with the geographic location of clusters of birth and menarche residences identified previously [14], we found that the size and location of these areas are about the same as clusters of menarche residences, but somewhat smaller than the clusters of birth residences. Thus, it is more likely that the same individuals are in both clusters. For post-menopausal residences, no area exceeding the critical value was detected.

Next, we evaluated effects of other risk factors on the risk surfaces. To create age-adjusted risk surfaces, the standardized difference between case and control densities stratified by menopausal status and age groups was examined. Table 3 presents the variability of risk surfaces when stratified by menopausal status and age groups. While there were similar numbers of geographic areas greater than 2 SD regardless of menopausal status and age groups, we found areas (about 8 rectangular grids) greater than critical values only for residences for premenopausal women, aged 35–44, and the geographic location of those areas was identical to the areas identified in Figure 3. In addition, we evaluated the effects of one known risk factor, nulliparity, on those spatial patterns of risk surfaces. Risk surfaces were examined in two groups, nulliparious women and those with at least one child. We observed no difference in spatial patterns of risk surfaces for these two groups.; for premenopausal women, there were seven and two geographic areas greater than critical values for nulliparious and parous women, respectively, but none was detected for either group of postmenopausal residences (data not shown).

**Table 3:** Standardized difference of residences for premenopausal and postmenopausal breast cancer cases and controls by age groups;

Premenopausal	Case (n = 1328)	Control (n = 2270)	Standardized differences	No. of areas^a ^> 2SD	No. of areas^a ^> critical values^b^
Ages 35–44 (n = 1889)	647	1242	-5.12–5.88	34	8
Ages 45–56 (n = 1709)	681	1028	-4.72–3.14	30	0

Postmenopausal	Case (n = 3484)	Control (n = 5616)	Standardized differences	No. of areas > 2SD	No. of areas > critical values

Ages 40–64 (n = 4916)	2115	2801	-3.52–3.77	49	0
Ages 65–79 (n = 4184)	1369	2815	-4.50–3.15	40	0

^a ^Areas refer to the rectangular grid overlaid on to the study area, ^b ^Critical values of 3.88 for premenopausal and 3.71 for postmenopausal residences.

Lastly, we were interested in evaluating temporal variability of risk surfaces; the standardized difference was obtained for each decade, for both pre- and post-menopausal residences, from the 1940s to 1990s (Table 4). While there was not much difference in the number of areas greater than 2 SD for both menopausal groups, we were able to find geographic areas greater than critical values in the 1960s through 1990s only for residences of premenopausal women. This is consistent with results from the above spatial analysis.

**Table 4:** Standardized difference of residences for premenopausal and postmenopausal breast cancer cases and controls by decades;

	Decades	Case	Control	Standardized differences	No. of areas^a ^> 2SD	No. of areas^a ^> critical values^b^
Premenopausal	1940s	36	86	-2.35–2.12	2	0
	1950s	222	461	-2.45–3.15	9	0
	1960s	341	633	-4.02–3.78	21	2
	1970s	514	1025	-3.89–3.96	35	2
	1980s	552	1005	-4.26–4.50	24	3
	1990s	457	723	-4.06–4.14	32	5

Postmenopausal	1930s	389	676	-2.51–2.62	24	0
	1940s	809	1253	-3.01–3.04	23	0
	1950s	1158	1968	-3.66–2.91	22	0
	1960s	1247	2072	-4.18–2.99	18	0
	1970s	1100	1839	-4.98–3.13	21	0
	1980s	960	1651	-3.84–2.85	23	0
	1990s	943	1542	-4.40–3.57	22	0

^a ^Areas refer to the rectangular grid overlaid on to the study area, ^b ^Critical values of 3.88 for premenopausal and 3.71 for postmenopausal residences.

## Discussion and conclusions

This study explored the use of kernel density estimation methods to identify spatio-temporal patterns of risk surfaces in a case-control study of breast cancer. We used standardized differences between case and control densities to produce risk surfaces. These risk surfaces were assessed for both pre- and postmenopausal breast cancer. We found a general tendency for spatial clustering of breast cancer cases, and observed stronger evidence of geographic clustering for pre-menopausal women than for postmenopausal women. Geographic areas greater than 2 SD of the standardized difference were identified among lifetime residences for pre- and postmenopausal women, but more rigorous testing showed such evidence only for premenopausal residences. We were able to pinpoint geographic areas with relatively higher case densities, and to assess temporal variability of risk surfaces.

This study focused on the investigation of breast cancer risk associated with lifetime residential history using GIS-based exploratory spatial analyses. Since environmental risk factors are of continuing interest in breast carcinogenesis, this approach may be more relevant in understanding the environmental etiology of breast cancer [15,16]. Residential location has often been used as a proxy for exposures, and the relationships between residential environment and breast cancer risk have been a focus in recent epidemiologic studies [17-19]. Although the role of clustering analyses remains controversial in scientific advances in our understanding of disease etiology [20,21], these GIS-based exploratory spatial analyses are well suited for environmental epidemiologic investigations; this study demonstrated that smoothed risk surfaces created by kernel methods are useful for large sets of data in space and time, but also when the form of cluster is not well defined. This method can be applied to other epidemiologic analyses. For example, this GIS-based spatial analysis can be effectively used in exposure analyses and assessment, as previously used in identifying people potentially exposed to environmental risk factors [22,23].

Given that these are exploratory methods, however, it is meaningful to compare the strengths and limitations of different approaches when applied to epidemiologic data. We have chosen the standardized difference approach to represent risk surfaces, as opposed to other methods (such as risk ratios) that can be used to create risk surfaces, because it is more easily able to handle the difficulties that arise with small densities. Unsmoothed risk surfaces are relatively easy to manipulate, but they are sensitive to geographic scales, while the ratio of case to control density results in unstable risk surfaces due to small number problems. We were able to reduce this problem in creating risk surfaces based on a standardized difference approach. The selection of optimal bandwidths in the application of kernel methods to cluster detection, and comparison of different types of bandwidths, such as adaptive kernel, will be a subject of future study [24].

It is important to note that current approaches to obtaining density surfaces of lifetime breast cancer risk are limited in several ways. First, we are using residential locations of breast cancer cases and controls; we obtained the difference in densities (risk surfaces) based on their residential location to identify areas with relatively higher case density. We do not know actual exposures and breast cancer risk associated with residential locations. However, this study provides evidence of differential exposure opportunities among cases and controls for further epidemiologic assessment, since spatio-temporal clustering of residential locations in a life course may be an indicator of differential exposure opportunities and the subsequent risk of breast cancer. Another limitation of this analysis is that we were unable to incorporate length of residence into the model. We were able to visualize risk surfaces with different weights based on the actual length of residence of each individual, but there were difficulties in incorporating this information into risk surfaces due to a substantial degree of variability in the length of residence. However, we observed spatial patterns consistent with those in Figure 3, despite the greater variability, when we visualized risk surfaces with length of residence information.

There is also need for cautious interpretation of these results due to the potential for selection bias inherent in the study design, including factors such as non-participation, and missing and excluded residential location. Although we had a relatively stable population and about 40% of study participants were lifetime residents in the study area, there may be different geographic patterns among those included and excluded groups. We excluded missing residential information and residential locations outside of the study area. In addition, we had a rate of non-participation of about 30% in the survey. However, in our earlier study [14], we found that characteristics of subjects in the study area were not different from characteristics of subjects with missing residential information and from subjects excluded due to residence outside of the study area, and that the geographic distribution of participants was not different from that of non-participants. Although we used the same methods for both cases and controls, and interviewers were blinded as to case and control status, there may be recall bias in the lifetime self-reported residential history. We validated the accuracy and reliability of lifetime residential history, especially earlier residences. Our finding was that reported residence information was generally correct. The greater problem was missing data; for this we conducted searches of historical records, as we have described earlier. We are now in the process of obtaining birth addresses from birth certificates for additional validation.

This study has significant implications for further studies on environmental exposure and breast cancer. We had previously found strong evidence of clustering of residence in early life, especially residence at birth and menarche [14]. In these analyses, we were able to show evidence of clustering of lifetime residence. In addition, we found breast cancer cases were more mobile than controls, and that premenopausal participants were more mobile than postmenopausal pariticipants. Average years at current residence was between 11 and 12 years for premenopausal participating cases and controls, compared to 22 and 23 years for postmenopausal participating cases and controls, respectively. Taking findings from this and our previous study together, it appears that examination of exposure opportunities in the past and across the lifespan may be critical for understanding environmental exposures related to breast cancer. Exploring spatio-temporal patterns of lifetime residential history may provide a link between this potential exposure and breast cancer risk. This study provides a more comprehensive analytical framework for the analysis of environmental exposures in relation to breast cancer by considering these components, such as migration and latency periods, and these spatio-temporal patterns of lifetime residential history may be a key to the understanding the actual relationships between environmental exposure and subsequent breast cancer risk.

To provide more accurate measures of personal cumulative exposures based on complete lifetime residential history, further studies should take into account the different effects of time periods or timing of exposures; construction of lifetime cumulative risk surfaces with different weights for exposure sensitivity at different points in time is a potential improvement and explanation of the methods employed to date. This approach may help to provide an answer to the question of where, when, and what kinds of exposures have influenced individuals' risk for a particular disease. Further, there has been recent development of a GIS-based framework to examine spatio-temporal patterns of lifetime residential history; the geospatial lifeline concept and space-time information system (STIS) approach is a good example of this [25-27]. We are currently testing the feasibility of similarity and difference measures of an individual's lifetime residential history using this case and control data, since it would be a powerful tool to analyze the personal environmental exposures associated with lifetime residential history.

In summary, we found evidence of clustering of lifetime residence for premenopausal cases relative to controls, and a substantial degree of spatio-temporal variability in the risk surfaces, thus providing a working hypothesis on breast cancer and environmental exposures. Geographic areas with higher case densities need further epidemiologic investigation for potential relationships between lifetime environmental exposures and breast cancer risk. Examination of lifetime residential history provided additional information on geographic areas associated with higher risk; limiting exploration of chronic disease clustering to current residence may neglect important relationships between location and disease. Further studies on the relationship between disease risk and environmental exposures associated with lifetime residential history should be replicated in other settings.

## Methods

### The Western New York Exposure and Breast Cancer Study (WEB Study)

Data from a population-based case-control study of breast cancer in western New York (the WEB Study) were used for our analyses. Participants were women, age 35–79 who were residents of Erie and Niagara counties, with no history of cancer other than non-melanoma skin cancer; cases were women with incident, primary, pathologically confirmed breast cancer, diagnosed during the period 1996–2001, and controls were randomly selected and frequency matched to cases on age, race, and county of current residence. Details of the WEB study, including selection, ascertainment, in-depth interview processes, have been described previously [14]. We collected lifetime residential histories for 1,166 cases and 2,105 controls, identified 20,240 lifetime addresses, an average of approximately 6 addresses for each individual, from participating cases and controls. Analyses were restricted to those residential locations within the two counties of study area.

### Geocoding of residential location

Geocoding of residential locations enables us to record each individual's locational information as *x *and *y *coordinates to be used in further spatial analyses. Address geocoding is a process that creates a theme based on the address data in a tabular form (event theme) and a reference feature theme (street map) to add point locations defined by the street address to the map. Matching depends not only on the quality of the reference theme, but also on the quality of the tabular data to be mapped. We used GDT/Dynamap 2000, an enhanced version of Topographically Integrated Geographic Encoding and Referencing system (TIGER), as a reference theme. In a study validating the positional accuracy of TIGER for the use in epidemiologic study [28], we found positional accuracy to be extremely high.

The overall matching rate for the 15,487 Erie and Niagara County residential locations was 82% (12,698); 91% of the matches were matched with complete information (good match), while about 9% of were estimated with partial information (interactive match) as detailed below. Geocoding success rates were lower for earlier residences, mainly due to more missing and partial information of earlier residences and changes in streets names and zip codes. However, it is important to note that there were few changes in street structure for this region during the time period of interest. There was, of course, addition of new streets, but existing streets were unchanged for the most part, and thus we found that the process of geocoding using current information was not inappropriate. We utilized various resources, including historic city directories with address information for residents, historical maps, and commercial address databases, to find missing residence information, and we developed several strategies to improve matching rates [14]. In addition, for residences where we had a known street name but no known street number and if the total length of the street was one kilometre or less, we estimated the residence location as the midpoint of the street.

### Kernel density estimation methods

Kernel density estimation methods have been used for disease mapping and for the detection of geographical location of clusters in epidemiologic settings [10,12,29]. A general form of the kernel *k *is defined as, , and by averaging over these individual kernel functions, we obtain the kernel density estimator,  ;



where *x *is the location for density estimation, *x*_*i *_is the observed point location, *n *is the number of points, and *h *is a smoothing parameter that regulates the degree of smoothness. Kernel functions are symmetric around zero and integrate to one. Both kernel and kernel density estimator are density functions; thus ∫*k*_x _(*x*)dx = 1 and ∫*f*_h_(*x*)dx = 1. For the two-dimensional kernel density estimator:



While the estimation of intensity for one point pattern may show patterns of high and low risk areas, this is of limited utility in epidemiologic applications because it may largely reflect the pattern of population distribution [30]. The ratio of case to control density can be more effectively used in epidemiologic settings [9,31]. To estimate the ratio of the two density estimates, the ratio of case to control density,  is used, where  and  are estimates of the intensity for cases and controls, respectively. Smoothed risk surfaces using the ratio or log ratio of case to control density can be less effective with small sample sizes; it is possible to have spurious, high risk areas in the application of the ratio of densities when the value of the control density is too small.

The relative difference between two densities provides an alternative way to assess the spatial variability of risk surfaces. Using the square root variance stabilizing transformation and the standardized difference, this measure allows us to identify areas with differences between case and control density exceeding two standard deviations [32]. The standardized difference between case and control densities is obtained by taking the square root of the case density minus the square root of the control density, and dividing by the standard deviation of the difference between the densities.



where 

### Analytical procedures

The first step in creating smoothed risk surfaces using kernel methods was to create reference grids and overlay the study area with them. We obtained the smoothed intensity for both cases and controls by calculating the distance between each point on the reference grid and the locations of breast cancer cases and controls. We used the quartic kernel to estimate the intensity of points at each grid point, although the choice of kernel type is not crucial as long as the kernel is symmetrical [33]. We applied equal, fixed bandwidths for both cases and controls using equation (1) because the objective is to describe overall patterns of the underlying spatial distribution [9]. The risk surface was obtained by forming the difference in densities based on equation (2).

The above analyses were repeated with varying bandwidths because the choice of appropriate bandwidth is one of the primary concerns of the kernel method. Although a subjective choice made from a range of values is commonly used [33], selections of bandwidth were made here on the basis of several factors. To avoid subjectivity, we first began with the commonly used optimal bandwidth designed to minimize the estimated mean square error [34]. Other established methods, such as cross validation, were tried and these resulted in small bandwidths because of the large sample size [34,35]. In addition, to take into account the spatial distribution of point patterns and to avoid problems associated with fixed bandwidths, we initially selected bandwidths based on the average distance among points. Since the size of the study area is approximately 30 miles in width and 60 miles in length, we selected a one-mile radius as an initial bandwidth of the kernel, with a range of 0.5 to 10 miles. In summary, we searched over a range of bandwidths and ultimately chose a two-mile bandwidth as a balance between over-smoothing and under-smoothing. Increasing bandwidth implies increasing the amount of smoothing in the estimate. A larger bandwidth results in very smooth density surfaces, while too small a bandwidth produces noisy density estimates. These issues are important in applications of our case-control data; the geographic distribution of cases and controls is dependent on the population distribution, which is greatly concentrated in urban areas and is sparser in rural areas. Thus, the use of a small bandwidth less than two miles resulted in an unrecognizable pattern of density.

Risk surfaces based on the ratio and difference in density surfaces between cases and controls were implemented in a GIS and S-Plus environment [36]. In addition to finding the risk surface associated with the standardized difference between densities, we tested the significance of the difference surfaces between cases and controls by Monte Carlo simulation. Under the null hypothesis of constant risk in the study area, we obtained critical values. We first randomly assigned case and control status to each of the case and control locations, based on the proportion of cases and controls among the set of cases and controls. Then we obtained the 95th percentile for the maximum difference between case and control densities from 999 simulations.

## Authors' contributions

All authors have participated fully in the conduct of this study, the acquisition of data, the analysis and/or the preparation of the manuscript in a substantial manner. DH performed the statistical analysis and drafted the manuscript; PAR and JLF provided critical review and input. JLF, JEV, MAB, JN, PM and MT participated in the design of the study, participated in interpretation, as well as in data acquisition efforts. All authors read and approved this manuscript.
